# GhoMR: Multi-Receptive Lightweight Residual Modules for Hyperspectral Classification

**DOI:** 10.3390/s20236823

**Published:** 2020-11-29

**Authors:** Arijit Das, Indrajit Saha, Rafał Scherer

**Affiliations:** 1 Tata Consultancy Services Limited, Kolkata 700 091, India; ad81952@gmail.com; 2 Department of Computer Science and Engineering, National Institute of Technical Teachers’ Training and Research, Kolkata 700 106, India; 3 Institute of Computational Intelligence, Czȩstochowa University of Technology, 42-201 Czȩstochowa, Poland

**Keywords:** convolutional neural network, deep learning, feature extraction, hyperspectral image classification, multi-receptive module, remote sensing

## Abstract

In recent years, hyperspectral images (HSIs) have attained considerable attention in computer vision (CV) due to their wide utility in remote sensing. Unlike images with three or lesser channels, HSIs have a large number of spectral bands. Recent works demonstrate the use of modern deep learning based CV techniques like convolutional neural networks (CNNs) for analyzing HSI. CNNs have receptive fields (RFs) fueled by learnable weights, which are trained to extract useful features from images. In this work, a novel multi-receptive CNN module called GhoMR is proposed for HSI classification. GhoMR utilizes blocks containing several RFs, extracting features in a residual fashion. Each RF extracts features which are used by other RFs to extract more complex features in a hierarchical manner. However, the higher the number of RFs, the greater the associated weights, thus heavier is the network. Most complex architectures suffer from this shortcoming. To tackle this, the recently found Ghost module is used as the basic building unit. Ghost modules address the feature redundancy in CNNs by extracting only limited features and performing cheap transformations on them, thus reducing the overall parameters in the network. To test the discriminative potential of GhoMR, a simple network called GhoMR-Net is constructed using GhoMR modules, and experiments are performed on three public HSI data sets—Indian Pines, University of Pavia, and Salinas Scene. The classification performance is measured using three metrics—overall accuracy (OA), Kappa coefficient (Kappa), and average accuracy (AA). Comparisons with ten state-of-the-art architectures are shown to demonstrate the effectiveness of the method further. Although lightweight, the proposed GhoMR-Net provides comparable or better performance than other networks. The PyTorch code for this study is made available at the iamarijit/GhoMR GitHub repository.

## 1. Introduction

Hyperspectral images (HSIs) are image cubes where each pixel is measured as one near-continuous spectrum. Unlike RGB images, HSIs have hundreds of spectral bands, containing knowledge regarding wavelengths beyond the visible spectrum. These cubes contain both spatial and spectral information, which can be widely utilized in remote sensing for analyzing a scene of interest. Hyperspectral imaging also finds its applications in agriculture [1], forestry [2,3], archaeology [4], medical analysis [5], food quality control [6], military defense [7], forensics [8], and several other domains as well. Thus, research in HSI processing and analysis is growing rapidly, and several studies have been published in past years for the same. Often, the high spectral dimensionality of an HSI poses a challenge in the analysis due to noise and high computation costs. Earlier, algorithms like independent component analysis (ICA) [9], principal component analysis (PCA) [10], and linear discriminant analysis (LDA) [11] were used to deal with this. Recently, more advanced dimension reduction techniques [12,13,14] and band selection methods [15,16,17] have been found to address the same. An HSI is also subject to mixed pixels, i.e., a pixel can contain mixtures of spectra from different components (also called endmembers). This occurs either due to the low spatial resolution of the sensors or due to multiple scattering and intimate mixing effects. Thus, spectral unmixing is done, which involves retrieving all or some of the endmembers and estimating their fractional abundances in each of the mixed pixels. In recent years, several techniques [18,19,20] have been proposed, which have shown satisfactory results in hyperspectral unmixing. Similarly, HSI classification is another widely-concerned task in hyperspectral imaging, which this manuscript addresses. HSI classification is the process of assigning a class for every pixel in an image, based on its spectral and spatial features. Early researches on HSI classification mostly focused on utilizing shallow hand-crafted techniques [21,22]. Some of these techniques [23] utilize local covariance matrix representation to extract the correlation between the spectral bands, which are then used by machine learning algorithms, like support vector machine (SVM) [24] for HSI classification. Along with spectral methods, spatial feature extraction techniques like mathematical morphological transformations [25] and composite kernel learning [26,27] are also used. 3D wavelets [28] and 3D Gabor filters [29] are also efficient methods for extracting spatial features from HSIs. Other techniques [30,31,32] involving sparse representations are also developed to exploit the spatial contextual knowledge in HSIs.

Although the methodologies discussed above have effectively addressed HSI classification, they are capable of extracting only a limited set of features, deficient in useful information. This limitation has inspired deep learning computer vision (CV) algorithms to replace these shallow hand-engineered techniques. This evolution is discussed in details in a recently published comparative study [33] between the shallow techniques and learning-based algorithms. Convolutional neural network (CNN) is one of the widely used deep learning algorithms for HSI classification. A CNN is driven by receptive fields (RFs), which use trainable filters to extract features from HSIs. These filters have randomly initialized weights, which automatically update while training to extract necessary information. This self-learning potential gives CNN robustness and superior discriminative ability than shallow methods to distinguish between various HSI pixels. Besides HSI classification, CNN architectures proposed in recent years have also revolutionized other domains of CV. AlexNet [34], proposed in 2012, is one of the founding architectures for image classification on the ImageNet [35] dataset. Several architectures like VGGNet [36], GoogleNet [37], ResNet [38], DenseNet [39] and SENet [40] followed. Methods have been proposed to tackle other CV tasks—R-CNN [41], fast R-CNN [42], faster R-CNN [43], YOLO [44] and SSD [45] for object detection, mask R-CNN [46], SegNet [47], FCN [48] and U-Net [49] for image segmentation, RCCNet [50] for colon cancer classification, etc.

For HSI analysis, several CNN-driven architectures are proposed in recent years. Some simple networks use 2D-CNN [51] and 3D-CNN [52]. Other networks like deformable CNN [53], super-resolution-aided CNN [54] and Two-CNN [55] use variations of 2D-CNN, while multi-scale 3D-CNN (M3D-CNN) [56], 3D-LWNet [57] and spectral-spatial residual network (SSRN) [58] use 3D-CNN-based approaches. HybridSN [59], another state-of-the-art architecture, uses a sequential fusion of both 2D and 3D CNNs to extract joint spectral-spatial information. Dual-path network (DPNet) [60], convolutional feature fusion network [61] and deep feature fusion network [62] are other fusion-based strategies for HSI classification. FuSENet [63], which uses squeeze-and-excitation modules [40], applies fusion within a single residual block. Unlike SENet, which uses global average pooling (GAP) for squeeze operation, FuSENet uses a fusion of GAP and global max-pooling (GMP) for the same. Although these methods have excelled tremendously in HSI classification, they have fairly heavy architectures, owing to a large number of trainable parameters. Since CNNs are significantly machine-dependent, these architectures require expensive GPUs and hardware to train and store them.

The above shortcoming in earlier works inspired us to propose the multi-receptive lightweight residual block called GhoMR. A singular GhoMR uses a complex strategy inspired by Res2Net [64] to extract information from HSI data. Each module contains multiple RFs, where each RF extracts features in a hierarchical fashion using information from other RFs in the same module. These RFs are connected with residual-like connections. However, with an increase in complexity, the number of learnable weights increases. Thus, to ensure a lightweight architecture, the Ghost module (GM) is used as the basic building unit. A single receptive layer of a CNN has multiple convolutional kernels which generate several feature maps. Research has shown [65] that many of these feature maps are similar and can be easily constructed by transforming other features. GMs take advantage of this feature redundancy in CNNs. Inside a GM, a very limited number of features are extracted from the input using a convolutional layer. Then, more features are generated from the existing ones using cheap linear operations on them. This strategy reduces the number of parameters, giving rise to a lightweight feature extraction module. The GM was first used in GhostNet [65], published in CVPR 2020, and later it became a backbone for many methods. Recently, an architecture based on GM called Improved GhostNet [66] was used for remote sensing classification as well. However, the proposed GhoMR is the first to use GM on HSIs. Stacking four such GhoMR modules, a classification network called GhoMR-Net is constructed, which is tested on three benchmark datasets and compared with state-of-the-art architectures.

The main contributions of this research can be summarized as follows:

1. A novel lightweight multi-receptive feature extraction module called GhoMR is proposed for HSI classification,
2. A GhoMR utilizes complex feature extraction strategy using several internal RFs, connected in a residual fashion,
3. To reduce the number of trainable parameters, Ghost modules are used, which uses low-cost transformations to address feature redundancy in CNNs,
4. An architecture called GhoMR-Net is designed using multiple GhoMR blocks to perform experiments on three public HSI datasets,
5. Comparisons are shown, which verifies that the proposed GhoMR gives better or comparable results than state-of-the-art techniques.

The rest of the paper is organized as follows. Section 2 describes the proposed methodology, Section 3 describes the datasets used and discusses the experiments, comparisons, and visualizations performed on them, while Section 4 concludes our research.

## 2. Methodology

### 2.1. Brief Description of Ghost Modules

CNNs are driven by receptive kernels or filters having randomly initialized weights. These kernels traverse an input (image or feature maps) and perform element-wise multiplication with underlying pixels, followed by summation to extract features. This operation is termed as convolution. During training, sufficient examples are fed, and along with many iterations, these weights are updated using backpropagation, as the network learns to generalize over unseen examples. However, CNN architectures use several kernels to extract a wide variety of feature maps. This increases the cardinality of trainable weights, thus demanding heavy computational costs and expensive hardware to train and store them.

Let $I\in R^{W\times H\times C}$ be the input to a single convolutional block, where *W* and *H* are the spatial dimensions, while *C* is the number of channels. To extract a unique feature map $y_{i}$ from *I*, a kernel $k_{i}\in R^{s\times s\times C}$ is used to perform the convolution, where s<W and s<H. The convolution operation can be represented as

(1) $$y_{i}=Conv_{s\times s}\left(I\right)$$

Similarly, a set of $C^{\prime }$ kernels $\left\{k_{1},k_{2},k_{3},\ldots ,k_{C^{\prime }}\right\}$ is used to generate different feature maps, which are stacked to produce a feature block $Y\in R^{W^{\prime }\times H^{\prime }\times C^{\prime }}$, which becomes the input for another set of kernels. This total operation involves $s\times s\times C\times C^{\prime }$ number of parameters, which can be as large as hundreds or thousands, owing to large values of *C* and $C^{\prime }$. Thus, to reduce parameters, the number of kernels, $C^{\prime }$ must be optimized (assuming that *C* is constant). Prior research has shown that many feature maps derived by these kernels are similar to each other. So, these can be generated by mutating the existing ones, rather than using separate kernels. To exploit this redundancy, the Ghost module (GM) [65] was recently invented.

A GM reduces the cardinality of kernels while keeping a minimal loss of information at the same time. Feature extraction in a GM is done in two steps:

1. The first step involves simple convolutional operations as described above. Keeping all hyper-parameters constant, $C^{\prime \prime }$ kernels are used to generate a set of intrinsic feature maps $Y^{\prime }=\left\{y_{1}^{\prime },y_{2}^{\prime },y_{3}^{\prime },\ldots ,y_{C^{\prime \prime }}^{\prime }\right\}$, where $C^{\prime \prime }<<C^{\prime }$. As a result, the total number of parameters in the network reduces to $s\times s\times C\times C^{\prime \prime }$.
2. The reduction of parameters leads to the loss of significant information. To make up for the remaining $C^{\prime }-C^{\prime \prime }$ features, new feature maps are derived from each of the existing features by performing *T* low-cost operations (Ghost transformations) on them. These derived features are called Ghost features. This equation can be represented as
  (2) $$y_{ij}^{g}=\theta_{ij}\left(y_{i}^{\prime }\right),$$
  where $y_{i}^{\prime }$ is the *i*th feature map in $Y^{\prime }$ and $\theta_{ij}$ is the *j*th linear operation deriving a Ghost feature $y_{ij}^{g}$ from $y_{i}^{\prime }$. Thus, $1\leq i\leq C^{\prime \prime }$ and 1≤j≤T. Among the *T* Ghost transformations applied on $y_{i}^{\prime }$, one operation $\theta_{i1}$ is kept as identity operation to retain the original feature map. The remaining T−1 operations generates the ghost features. Thus, now a total of $C^{\prime \prime }\times T$ features are generated, such that $C^{\prime \prime }\times T∼C^{\prime }$.

**Figure 1:**
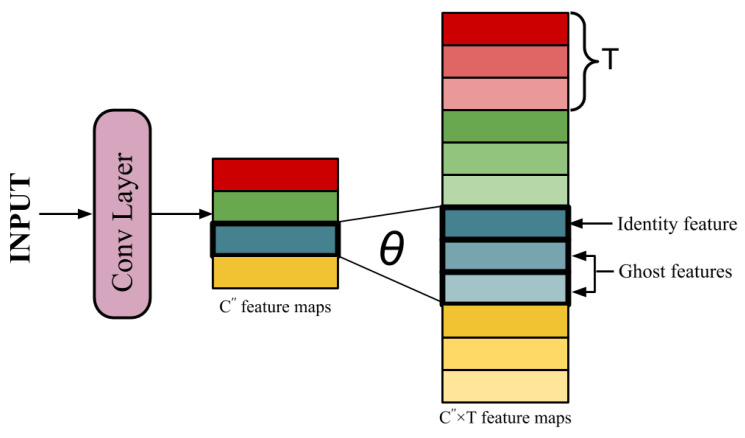
An illustration of the Ghost module.

Figure 1 shows a simple illustration of the Ghost module. For the transformation function θ, convolutional filters of size $K_{T}\times K_{T}$ are used instead of hand-crafted low-cost linear operations. These filters are called Ghost filters. This is done to utilize the learning capability of convolution operation to perform the most appropriate transformations. Moreover, it gives the flexibility to experiment with different values for $K_{T}$, since the kernels of different spatial dimensions extract different types of features. Note that the computational complexity of θ is much less than ordinary convolution, a detailed analysis of which is given in the founding manuscript [65].

### 2.2. GhoMR—Proposed Multi-Receptive Module for HSI Classification

**Figure 2:**
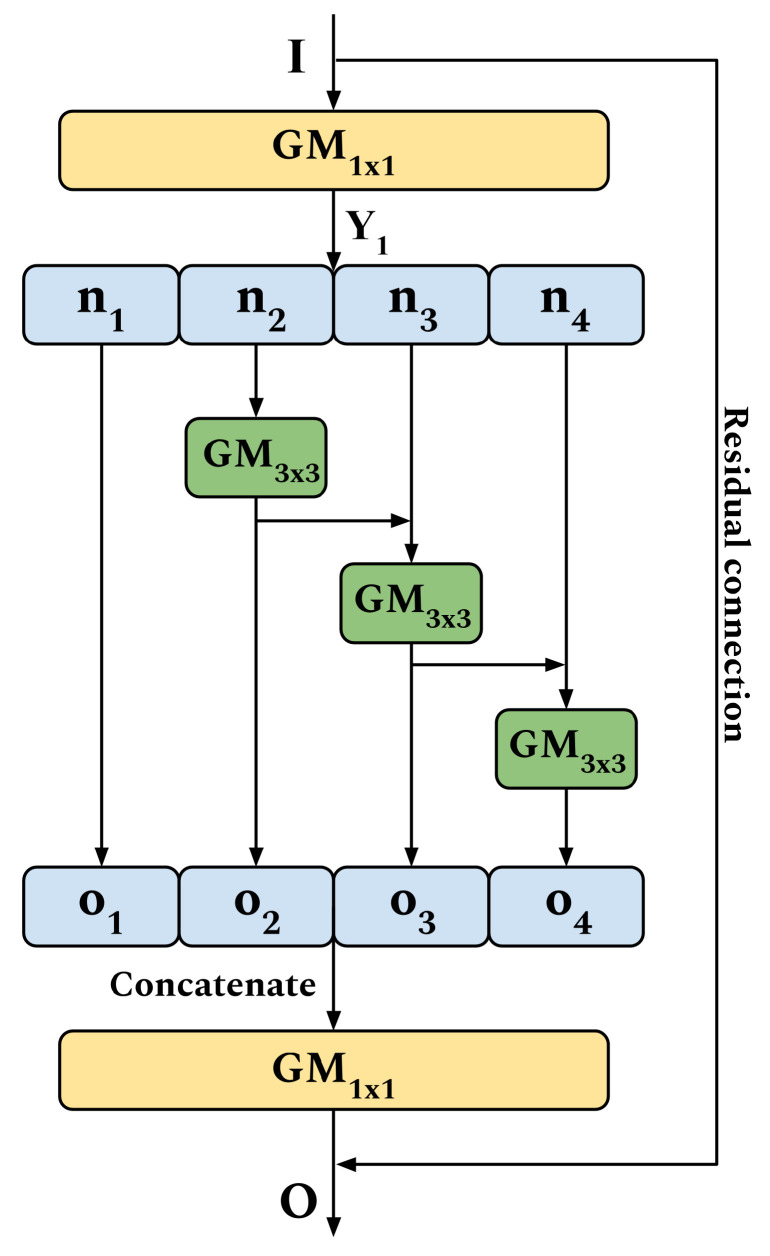
Proposed GhoMR module.

Figure 2 shows the diagram of a single GhoMR module, which is the proposed backbone for HSI classification. A GhoMR uses multiple internal GMs to extract features in a residual hierarchical fashion. This strategy is inspired by Res2Net [64] and is useful for extracting complex details from the HSI cube. Let the input for an arbitrary GhoMR module be $I\in R^{W\times H\times C}$, where W, H, and C are the width, height, and channels respectively. Feature extraction from this cube is done in three steps:

1. At first, a GM using 1×1 kernels is used to extract the feature block $Y_{1}\in R^{W\times H\times C}$.
  (3) $$Y_{1}=GM_{1\times 1}\left(I\right)$$
  Note, these 1×1 kernels are not the Ghost filters, but are used to generate the original feature maps. For the Ghost filters, experiments with different sizes ($K_{T}$) are performed, which is discussed in Section 3.
2. In the next step, the *N* feature maps of $Y_{1}$ are split into four subsets, denoted by $n_{i}$, where 1≤i≤4. Except $n_{1}$, each subset is passed through a 3×3 GM. The output of the previous GM, $o_{i-1}$ is fused hierarchically using element-wise summation with the current subset $n_{i}$, to produce the set of features $o_{i}$. The equations supporting this operation are
  (4) $$o_{i}=\left\{\begin{array}{cc} n_{i} & \mathrm{for}\;i\;=\;1\\ GM_{3\times 3}\left(n_{i}\right) & \mathrm{for}\;i\;=\;2\\ GM_{3\times 3}\left(n_{i}+o_{i-1}\right) & \mathrm{for}\;i\;=\;3,\;4 \end{array}\right.,$$
  where + refers to element-wise summation. Note, the GM for the first split $n_{1}$ is omitted in order to reuse features and reduce parameters in the module.
3. Finally, the output maps $o_{1}$, $o_{2}$, $o_{3}$ and $o_{4}$, are concatenated on their depth to form a singular feature block containing all the information. This is further passed through a 1×1 GM and fused with input *I* through a residual connection to produce the final output *O*. This operation is expressed as
  (5) $$O=GM_{1\times 1}\left(o_{1}⊕o_{2}⊕o_{3}⊕o_{4}\right)+I,$$
  where ⊕ refers to concatenation and + denotes element-wise summation.

## 3. Experiments and Discussion

### 3.1. Datasets

The proposed methodology is evaluated on three public HSI datasets (http://www.ehu.eus/ccwintco/index.php/Hyperspectral_Remote_Sensing_Scenes). The description of these datasets are given as follows:

1. Indian Pines (IP)—The images in this dataset were collected in 1992, over the Indian Pines test site in north-western Indiana using the AVIRIS [67] sensor. The HSI cube has a spatial dimension of 145×145 pixels with 224 spectral bands in the wavelength range of 400 to 2500 nm, among which 24 bands corresponding to regions of water absorption were eliminated. Among the 21,025 pixels, 10,249 are annotated with ground truth from a set of 16 different vegetation classes.
2. University of Pavia (UP)—This dataset was acquired in 2001, over the university campus at Pavia, Northern Italy, using the ROSIS sensor. It has a spatial dimension of 610×340 pixels and 103 spectral bands in wavelength between 430 to 860 nm. The ground truth is a set of 9 urban land-cover classes, and approx. 20% of the total 207,400 pixels are annotated with this information.
3. Salinas Scene (SA)—This dataset was collected over Salinas Valley, California, in 1998 using the AVRIS sensor. The spatial dimension is 512×217 pixels and the spectral information is encoded in 224 bands with a wavelength in the range of 360 to 2500 nm. Similar to IP, 20 spectral bands due to water absorption are discarded. The ground truth contains 16 different classes from vegetables, bare soils, and vineyard fields.

### 3.2. Experimental Protocols

**Figure 3:**
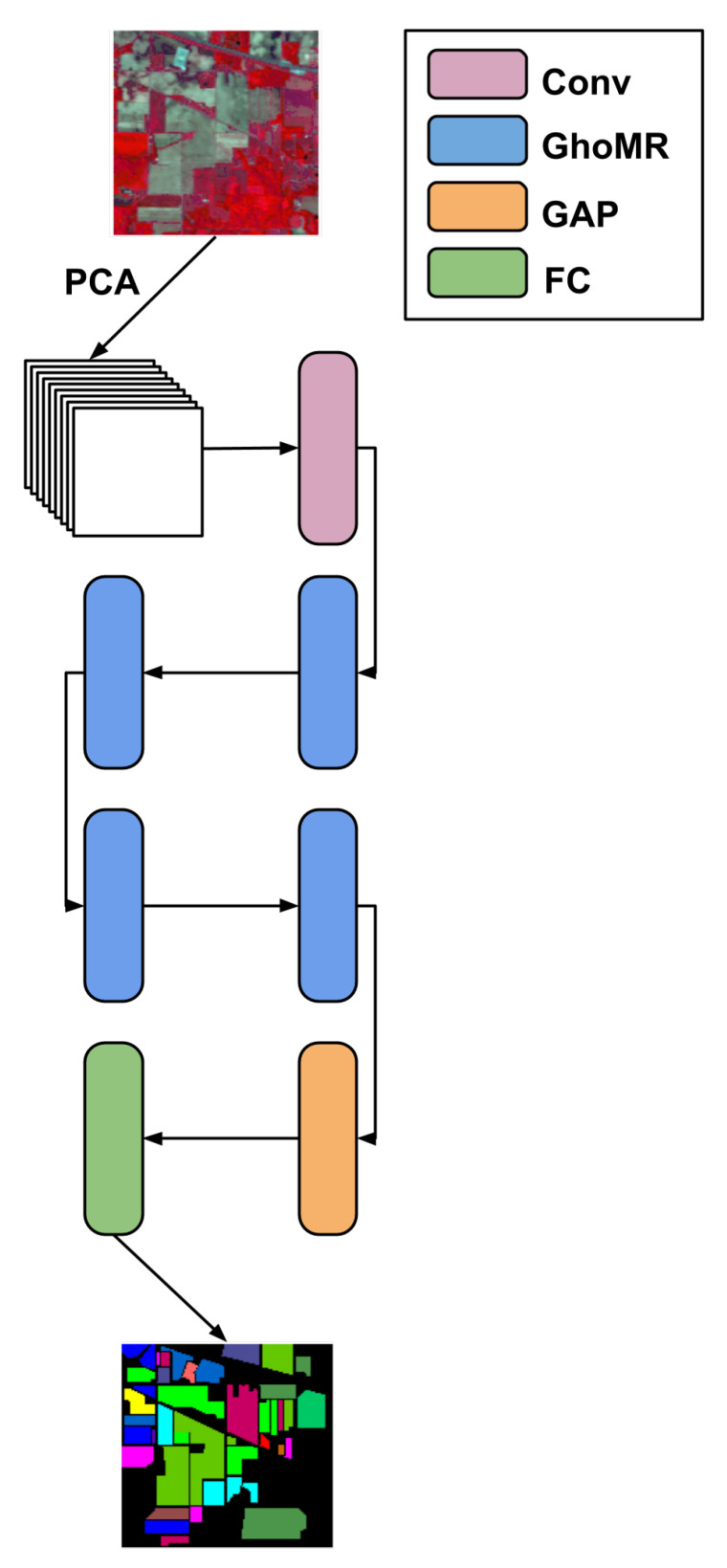
GhoMR-Net−Proposed HSI classification network.

Using several GhoMRs, a network called GhoMR-Net is proposed as shown in Figure 3. At first, the input is fed to a simple convolutional layer of 24 kernels. The output is then passed through a series of four GhoMR modules, which produces 24, 36, 48, and 60 feature maps, respectively. Inside each GhoMR, the first 1×1 GM generates 48 feature maps from the input, which is split into four parts, having 12 features each. The 3×3 GMs operating on each split ($n_{i}$) extract 12 feature maps, which are concatenated again into a single block of size 48. This block is fed to the final 1×1 GM, which outputs the set of features for the next GhoMR block. To increase the efficiency, after every GM batch-normalization [68] and ReLU activation is used. On the extracted features from the final GhoMR, global average pooling (GAP) [69] is performed and the resulting vector is fed to a fully-connected (FC) layer to output the class probabilities. The class with the maximum probability is the predicted class.

The above architecture is trained to classify each pixel of an HSI cube $C_{H}$. This 3D image cube has hundreds of spectral channels, containing redundant information. This makes classification difficult and increases computational costs. Thus, principal component analysis (PCA) is performed along the spectral axis. This PCA-reduced cube $C_{P}$ retains the spatial information and reduces the channels to *S*, where *S* is 30 for IP, and 15 for SA and UP respectively. Now, $C_{P}$ is divided into spatially overlapping 3D patches $D\in R^{W\times W\times S}$, where *W* is the spatial dimension of a patch. The ground-truth $Y_{T}\in R^{N_{C}\times 1}$ assigned to each patch is the same as that of the central pixel in the patch. These 3D patches are fed to the proposed GhoMR-Net, which outputs a vector $Y_{P}\in R^{N_{C}\times 1}$, where $N_{C}$ is the number of classes. The cross-entropy loss is then calculated between $Y_{T}$ and $Y_{P}$ and the network is trained to minimize this loss.

As discussed in Section 2, the GMs used in the GhoMR blocks have two hyperparameters—number of Ghost transformations (*T*) and spatial size of ghost filters ($K_{T}$). With an increase in *T*, less raw features are extracted from the input, and more are derived using Ghost operations, thus reducing the number of parameters. While a larger value of $K_{T}$ means a greater filter dimension, thus increasing trainable parameters in the network. Performance with different combinations of *T* and $K_{T}$ are discussed in the next subsection. Experiments with different spatial sizes (*W*) of input patches and different training ratios are also discussed. All the experiments are done using PyTorch 1.6.0 with CUDA 10.1 in the GPU environment of Google Colaboratory. The architecture is trained using Adam [70] optimizer for 100 epochs, keeping a batch size of 100 and a learning rate of 0.001. The code for this research is available at https://github.com/iamarijit/GhoMR.

To measure the performance, three standard evaluation metrics are used—overall accuracy (OA), average accuracy (AA), and Kappa coefficient. OA measures the total number of samples correctly classified in the test set, AA calculates the average of the class-wise accuracies and Kappa measures the degree of agreement between the ground-truth and predicted classification map. The OA, AA, and Kappa for each experiment are calculated five times and are written as mean ± std. Based on these metrics and the above-mentioned hyperparameters, five sets of analysis are carried out to demonstrate the classification potential and lightweight nature of the proposed GhoMR-Net:

1. First experiment calculates the class-wise accuracies, OA, AA, and Kappa for IP, UP, and SA datasets using 10% and 20% training data. The 3D spectral-spatial inputs have spatial dimensions 15×15 for all three datasets. The value of *T* and $K_{T}$ are kept 2 and 3 respectively.
2. In the second experiment, OA, AA, and Kappa are measured on the three datasets for different values of *T* and $K_{T}$, such that T∈{2,4} and $K_{T}\in \left\{3,5,7\right\}$. A comparative study between all the six combinations of *T* and $K_{T}$ is performed. This experiment is conducted on 10% training data with 3D input cubes of spatial dimension 15×15.
3. In the third experiment, the proposed architecture is compared with the following state-of-the-art techniques—SVM [24], 2D-CNN [51], 3D-CNN [52], M3D-CNN [56], Two-CNN [55], SSRN [58], HybridSN [59], SENet [63] (with global average pooling and max pooling) and FuSENet [63]. Comparisons are shown for both 10% and 20% training data, keeping input spatial dimension of 15×15.
4. The fourth experiment measures the OA, AA, and Kappa on lesser training data (5% and 3%) and smaller spatial dimensions (13×13 and 11×11) of input patches. The parameters *T* and $K_{T}$ are kept 2 and 3 respectively.
5. The final experiment demonstrates the effectiveness of GhoMR-Net using t-SNE visualization [71] and confusion matrices. Moreover, the number of trainable parameters in the network is compared with other state-of-the-art architectures.

### 3.3. Classification Results and Visualizations

**Figure 4:**
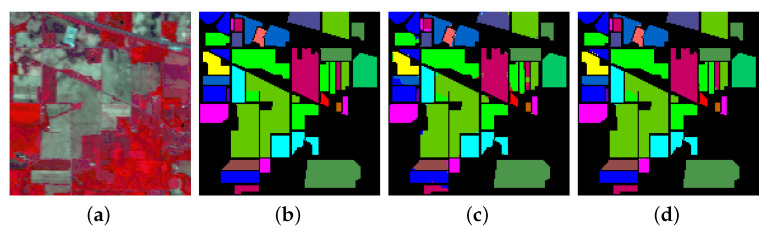
Classification maps for IP (**a**) False color image (**b**) Ground-Truth (**c**,**d**) Predicted maps for 10% and 20% training data, respectively.

**Figure 5:**
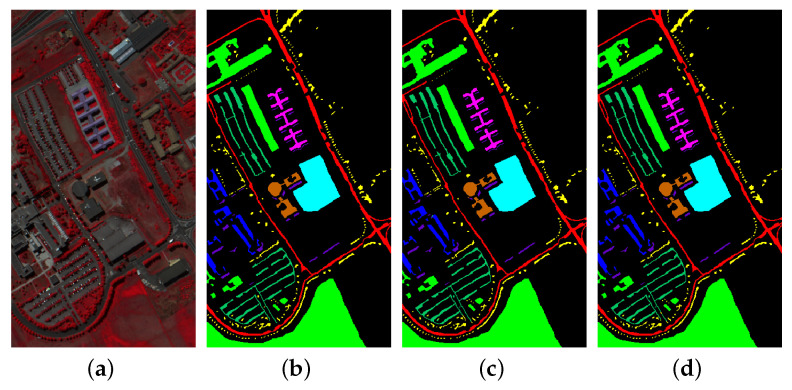
Classification maps for UP (**a**) False color image (**b**) Ground-Truth (**c**,**d**) Predicted maps for 10% and 20% training data, respectively.

**Figure 6:**
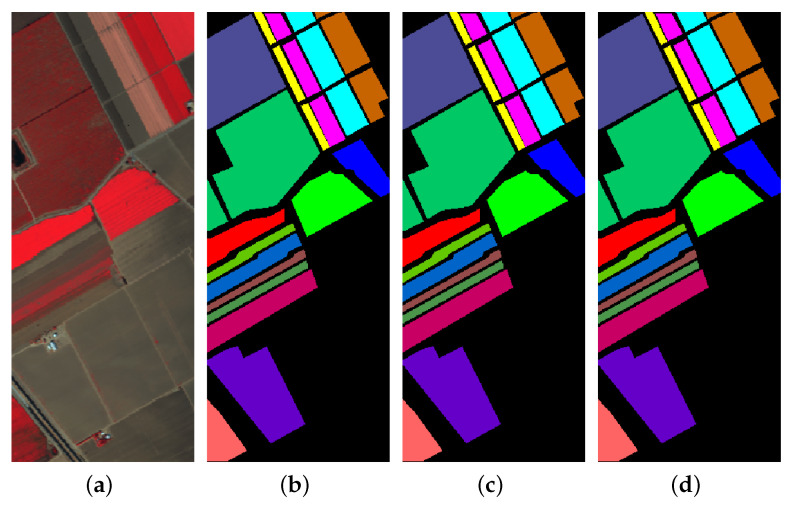
Classification maps for SA (**a**) False color image (**b**) Ground-Truth (**c**,**d**) Predicted maps for 10% and 20% training data, respectively.

The first experiment was conducted to calculate the class-wise accuracies for the three datasets, using hyperspectral inputs of spatial dimension 15×15. The results are shown in Table 1 and Table 2 for 20% and 10% training data, respectively. For each dataset, the first three columns contain class labels and data distribution (training and test samples), while the fourth column shows the accuracy (in percent %) for each class. The last four rows of the table represent the overall accuracy (OA), Kappa coefficient, average accuracy (AA), and training time for each experiment. For 20% training data, the OAs obtained are 99.54%, 99.90% and 99.99%, while on 10% data, it is 98.64%, 99.75% and 99.98% for IP, UP and SA, respectively. On IP, the proposed GhoMR-Net performs worse than SA and UP, which can be explained by fewer training examples and significant imbalance among the classes. To better understand the results, the ground-truth and predicted classification maps for IP, UP and SA are shown in Figure 4, Figure 5 and Figure 6, respectively.

In the second set of experiments, the dependence on the hyperparameters *T* and $K_{T}$ is explored. The OAs, Kappas, and AAs for different combinations of *T* and $K_{T}$ are given in Table 3. On IP and SA, the model performs best when T=2 and $K_{T}=3$, i.e., 2 ghost operations are used using 3×3 filters. Unlike IP and SA, the performance on UP increases when $K_{T}$ is increased. When $K_{T}$ is increased, the number of parameters increases. Since IP and SA have more classes (16) and fewer training samples per class (on an average), the tendency of overfitting increases with increasing $K_{T}$. Thus, performance on the test set decreases. Fixing the value of *T* and $K_{T}$ to 2 and 3 respectively, GhoMR-Net is compared with ten state-of-the-art techniques, using 10% and 20% training samples. The spatial window dimensions of the input are kept the same as the prior experiments. For IP, the method outperforms FuSENet, SSRN, and HybridSN with an increase in OA by 0.53%, 0.31%, and 0.07% respectively, on 20% training data. Improvements or comparable results are obtained on SA and UP as well, which is reported in Table 4. In spite of having very few parameters, the satisfactory classification results of GhoMR-Net can be explained by the multi-receptive feature extraction strategy of GhoMR modules.

**Figure 7:**
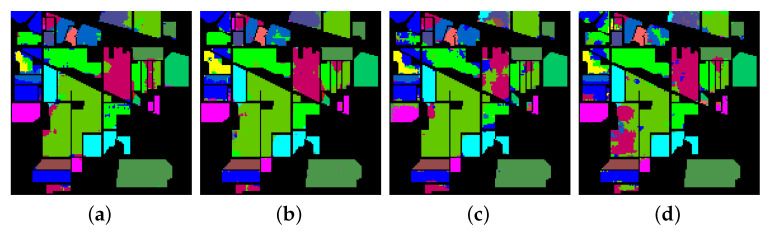
Predicted classification maps for IP with 11×11 and 13×13 input spatial size for (**a**,**b**) 5% training data and (**c**,**d**) 3% training data, respectively.

In the next experiment, the robustness of the approach and the influence of input spatial dimensions are explored. This is performed on lesser training samples, i.e., 5% and 3%, using inputs of spatial size 13×13 and 11×11. The OAs, AAs, and Kappas given in Table 5 show that performance deteriorates for all three datasets, which is expected. The classification maps for IP given in Figure 7 further verify it. It is observed, on increasing spatial size, the performance for IP and SA improves, since more spatial context is captured. However, in UP, as shown in Figure 5, the patches are short and discontinuous, unlike IP and SA. Thus, increasing spatial dimensions capture more noise, which reduces the classification accuracies.

**Figure 8:**
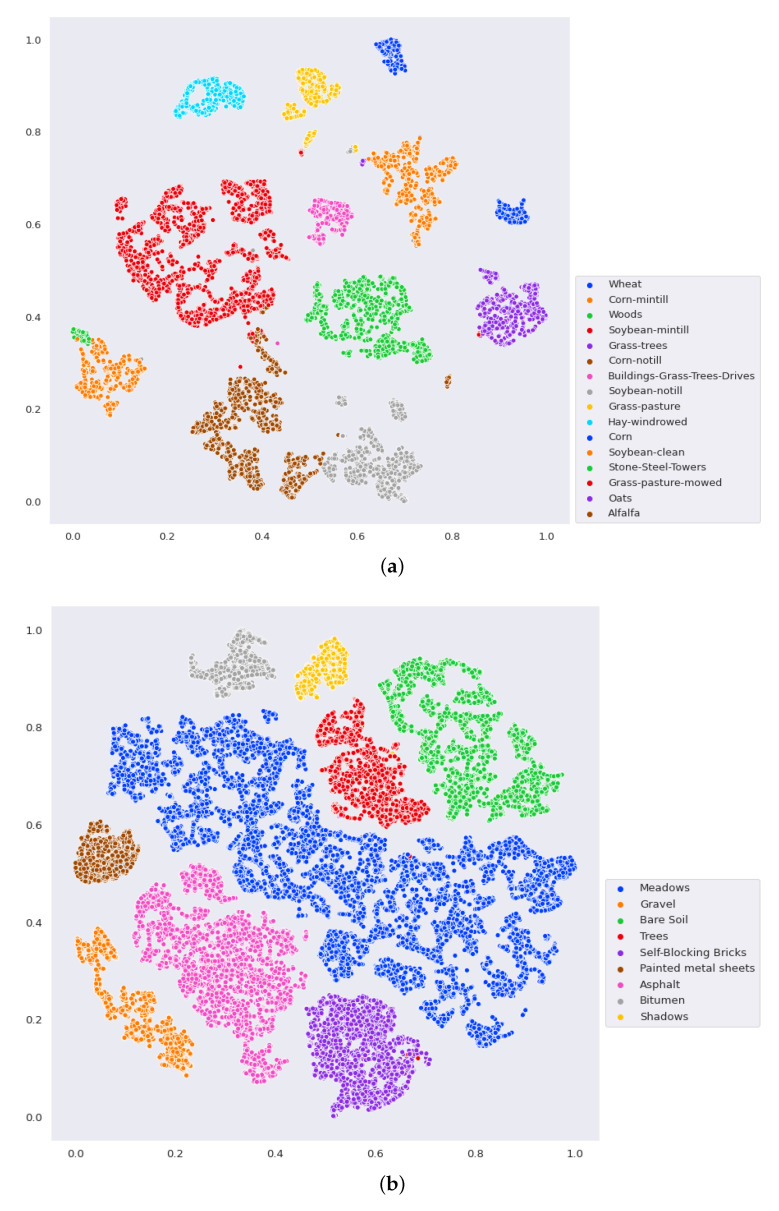

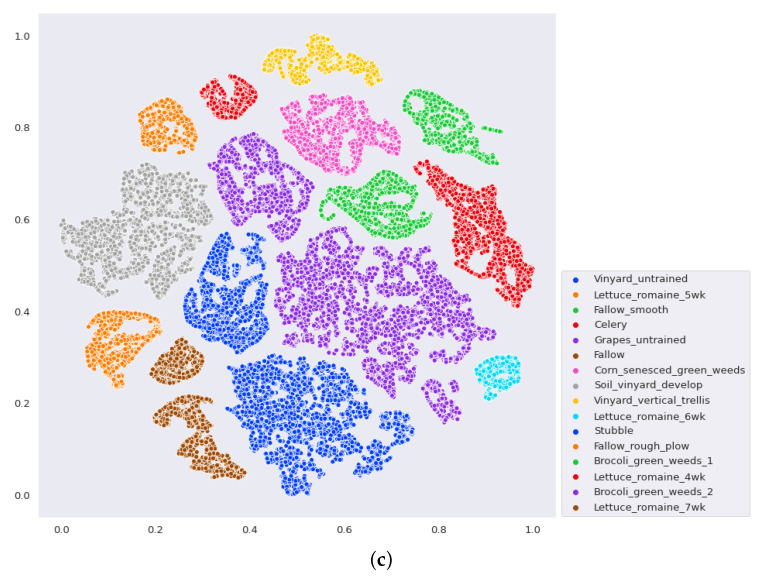
Visualization of extracted features via t-SNE where the 2D coordinates denotes the samples and the different colors represent different classes for the (**a**) IP, (**b**) UP, and (**c**) SA dataset.

**Figure 9:**
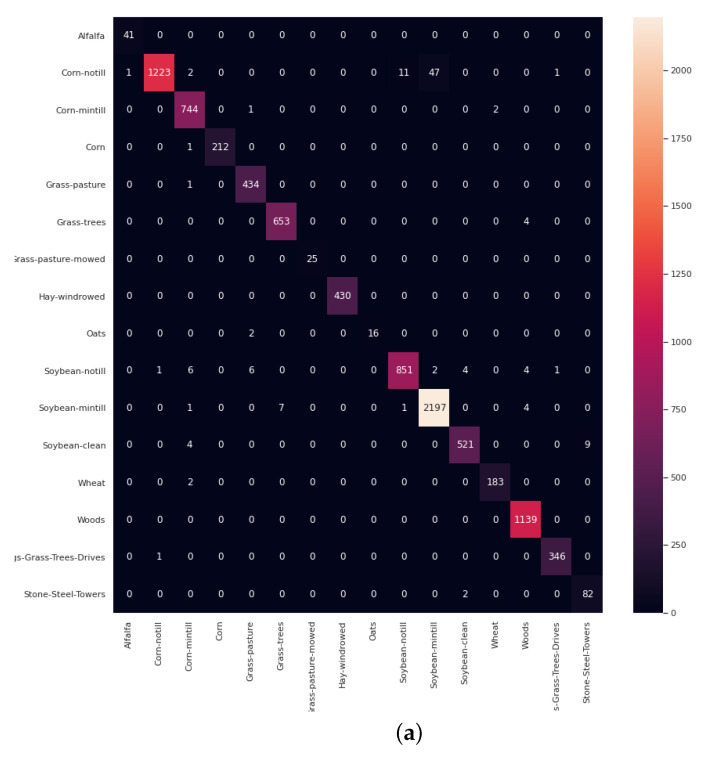

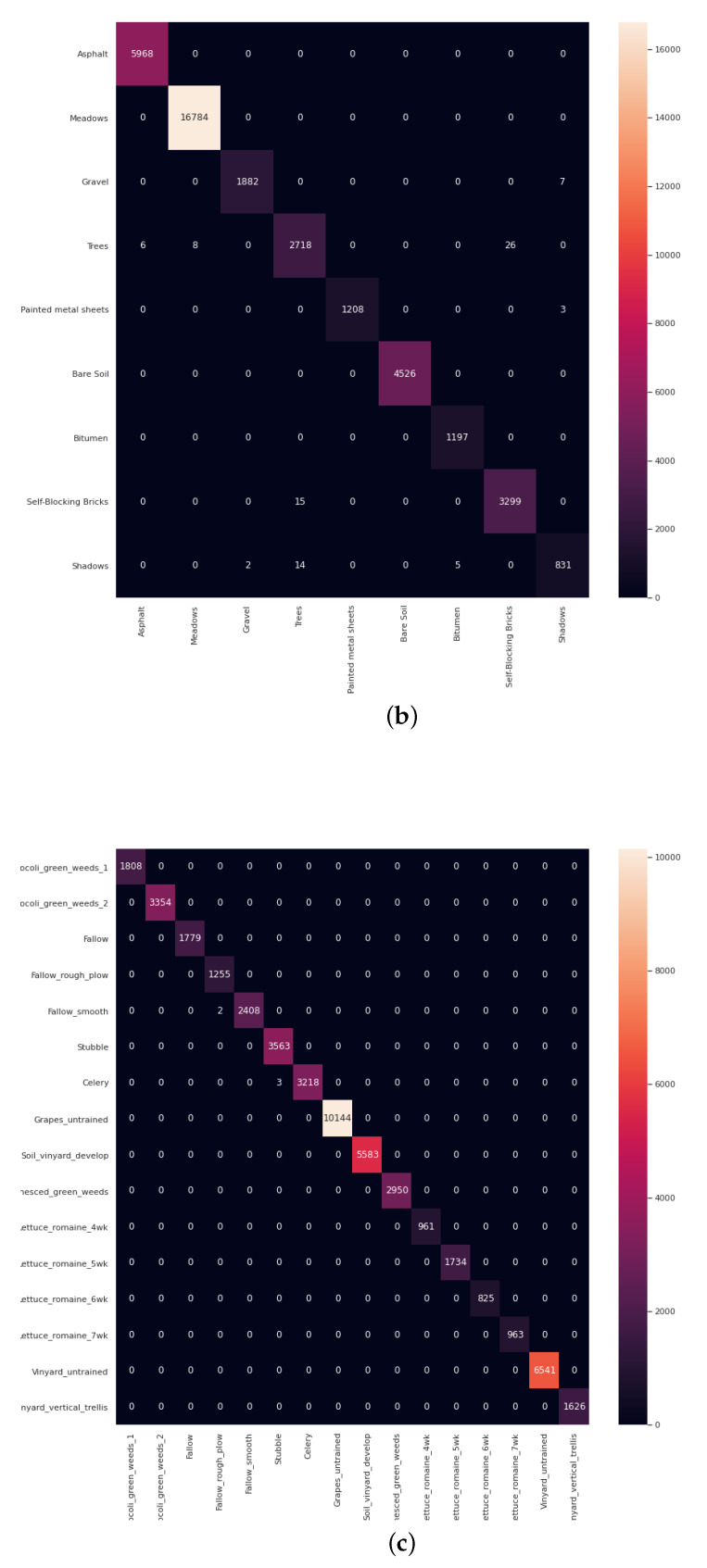
Confusion matrices obtained on 90% test samples for the (**a**) IP, (**b**) UP, and (**c**) SA dataset.

**Figure 10:**
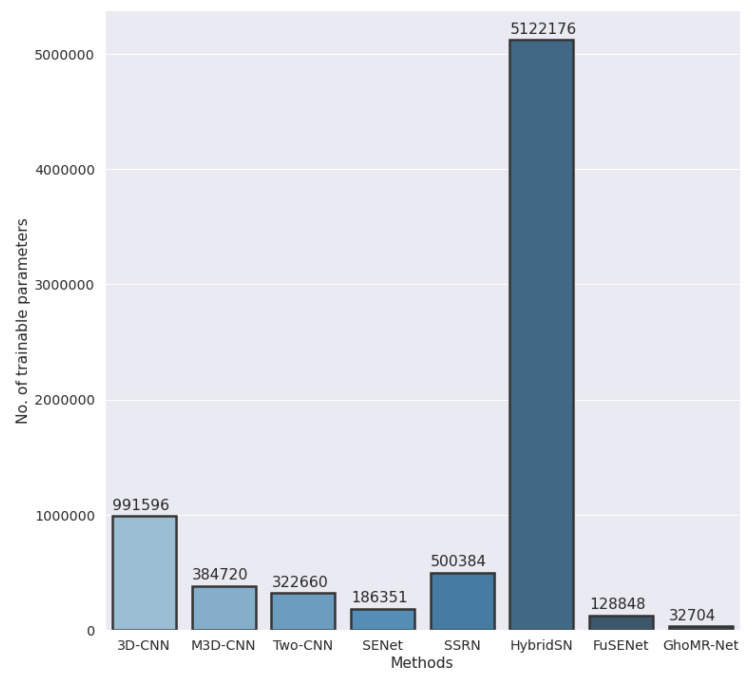
Number of trainable parameters in the proposed GhoMR-Net and other state-of-the art architectures.

Finally, a set of visualizations are performed to demonstrate the discriminative power of GhoMR-Net. The higher-dimensional features from the GAP layer of the network are extracted for each sample in the test set and are reduced to two-dimensional coordinates via t-SNE. These coordinates are plotted and shown in Figure 8 for the three datasets. It is clearly observed, that the features representing pixels having the same ground-truths form nearby clusters, which are represented by similar colors. Moreover, the confusion matrices are obtained on 90% test data and are given in Figure 9. Furthermore, the total number of trainable parameters is compared with seven above-mentioned architectures-3D-CNN [52], M3D-CNN [56], Two-CNN [55], HybridSN [59], SENet [63], FuSENet [63], and SSRN [58]. As shown in Figure 10, the proposed network has only 32,704 trainable parameters, which is much lesser than HybridSN, SSRN, and FuSENet having 5,122,176, 500,384, and 128,848 parameters, respectively.

## 4. Conclusions

In this study, a lightweight multi-receptive module called GhoMR is proposed for hyperspectral image (HSI) classification. It contains several internally connected receptive fields (RFs) to extract complex features from HSIs in a hierarchical approach. Unlike other approaches using convolutional layers, recently invented Ghost modules are used as RFs, which extracts hand-full features from the input and derives the remaining from existing ones. Using GhoMR blocks, a simple lightweight architecture called GhoMR-Net is designed to perform experiments on three standard datasets. The classification results are measured using three metrics and compared with other state-of-the-art techniques. Experiments with lesser training data and smaller input spatial sizes are also performed along with several visualizations and plots to understand the discriminative potential of the architecture better.

## Figures and Tables

**Table 1:** Data distribution along with class-wise accuracies, OAs, Kappas, AAs and training time on IP, UP and SA datasets, respectively, for 20% training data.

IP				UP				SA			
Name	Training	Test	Accuracy	Name	Training	Test	Accuracy	Name	Training	Test	Accuracy
Alfalfa	9	37	100±0.0	Asphalt	1326	5305	100±0.0	Brocoli_green_weeds_1	402	1607	100±0.0
Corn-notill	285	1143	98.81±0.3	Meadows	3730	14,919	100±0.0	Brocoli_green_weeds_2	745	2981	100±0.0
Corn-mintill	166	664	99.70±0.2	Gravel	420	1679	99.96±0.0	Fallow	395	1581	100±0.0
Corn	47	190	100±0.0	Trees	613	2451	99.00±0.2	Fallow_rough_plow	279	1115	99.98±0.0
Grass-pasture	97	386	99.79±0.2	Painted metal sheets	269	1076	99.93±0.1	Fallow_smooth	536	2142	99.86±0.2
Grass-trees	146	584	99.66±0.1	Bare Soil	1006	4023	100±0.0	Stubble	792	3167	100±0.0
Grass-pasture-mowed	6	22	100±0.0	Bitumen	266	1064	100±0.0	Celery	716	2863	100±0.0
Hay-windrowed	96	382	100±0.0	Self-Blocking Bricks	736	2946	99.72±0.1	Grapes_untrained	2254	9017	100±0.0
Oats	4	16	97.50±3.1	Shadows	189	758	99.82±0.1	Soil_vinyard_develop	1240	4963	100±0.0
Soybean-notill	194	778	99.54±0.2					Corn_senesced_green_weeds	656	2622	100±0.0
Soybean-mintill	491	1964	99.80±0.1					Lettuce_romaine_4wk	214	854	100±0.0
Soybean-clean	118	475	98.27±0.5					Lettuce_romaine_5wk	385	1542	100±0.0
Wheat	41	164	99.88±0.2					Lettuce_romaine_6wk	183	733	100±0.0
Woods	253	1012	100±0.0					Lettuce_romaine_7wk	214	856	100±0.0
Buildings-Grass-Trees-Drives	77	309	99.94±0.1					Vinyard_untrained	1453	5815	100±0.0
Stone-Steel-Towers	19	74	95.95±0.0					Vinyard_vertical_trellis	361	1446	100±0.0
OA	2049	8200	99.54±0.0	OA	8555	34,221	99.90±0.0	OA	10,825	43,304	99.99±0.0
Kappa			99.47±0.0	Kappa			99.86±0.0	Kappa			99.99±0.0
AA			99.30±0.2	AA			99.82±0.0	AA			99.99±0.0
Training time	3 min 34 s			Training time	13 min 50 s			Training time	17 min 52 s		

**Table 2:** Data distribution along with class-wise accuracies, OAs, Kappas, AAs and training time on IP, UP and SA datasets respectively for 10% training data.

IP				UP				SA			
Name	Training	Test	Accuracy	Name	Training	Test	Accuracy	Name	Training	Test	Accuracy
Alfalfa	5	41	98.54±2.0	Asphalt	663	5968	100±0.0	Brocoli_green_weeds_1	201	1808	100±0.0
Corn-notill	143	1285	96.45±0.8	Meadows	1865	16,784	100±0.0	Brocoli_green_weeds_2	372	3354	100±0.0
Corn-mintill	83	747	99.46±0.4	Gravel	210	1889	99.63±0.2	Fallow	197	1779	100±0.0
Corn	24	213	99.53±0.3	Trees	306	2758	98.61±0.2	Fallow_rough_plow	139	1255	99.97±0.1
Grass-pasture	48	435	99.54±0.3	Painted metal sheets	134	1211	99.9±0.1	Fallow_smooth	268	2410	99.85±0.2
Grass-trees	73	657	99.24±0.4	Bare Soil	503	4526	100±0.0	Stubble	396	3563	99.99±0.0
Grass-pasture-mowed	3	25	100±0.0	Bitumen	133	1197	100±0.0	Celery	358	3221	99.93±0.1
Hay-windrowed	48	430	100±0.0	Self-Blocking Bricks	368	3314	99.47±0.2	Grapes_untrained	1127	10,144	100±0.0
Oats	2	18	90.00±12.4	Shadows	95	852	96.38±0.6	Soil_vinyard_develop	620	5583	100±0.0
Soybean-notill	97	875	98.08±0.8					Corn_senesced_green_weeds	328	2950	100±0.0
Soybean-mintill	245	2210	99.28±0.2					Lettuce_romaine_4wk	107	961	100±0.0
Soybean-clean	59	534	95.73±3.0					Lettuce_romaine_5wk	193	1734	100±0.0
Wheat	20	185	99.46±0.5					Lettuce_romaine_6wk	91	825	100±0.0
Woods	126	1139	100±0.0					Lettuce_romaine_7wk	107	963	100±0.0
Buildings-Grass-Trees-Drives	39	347	98.90±0.9					Vinyard_untrained	727	6541	100±0.0
Stone-Steel-Towers	9	84	93.81±5.5					Vinyard_vertical_trellis	181	1626	100±0.0
OA	1024	9225	98.64±0.2	OA	4277	38,499	99.75±0.0	OA	5412	48,717	99.98±0.0
Kappa			98.45±0.3	Kappa			99.67±0.0	Kappa			99.98±0.0
AA			98.00±0.8	AA			99.33±0.1	AA			99.98±0.0
Training time	2 min 58 s			Training time	11 min 20 s			Training time	14 min 20 s		

**Table 3:** OAs, Kappas and AAs obtained for different values of *T* (no. of Ghost transformations) and $K_{T}$ (Ghost filter size) on IP, UP and SA datasets respectively (for 10% training data).

*T*	$K_{T}$	IP			UP			SA		
		OA	Kappa	AA	OA	Kappa	AA	OA	Kappa	AA
	3	98.64±0.2	98.45±0.3	98.00±0.8	99.75±0.0	99.67±0.0	99.33±0.1	99.98±0.0	99.98±0.0	99.98±0.0
2	5	98.51±0.2	98.30±0.2	98.26±0.2	99.77±0.0	99.70±0.0	99.42±0.1	99.97±0.0	99.97±0.0	99.96±0.0
	7	98.50±0.2	98.29±0.2	98.17±0.5	99.78±0.0	99.71±0.0	99.40±0.1	99.96±0.0	99.96±0.0	99.95±0.0
	3	98.19±0.3	97.94±0.3	97.67±0.9	99.72±0.1	99.64±0.1	99.26±0.1	99.98±0.0	99.97±0.0	99.97±0.0
4	5	98.12±0.4	97.86±0.5	96.80±0.8	99.80±0.0	99.74±0.0	99.47±0.1	99.97±0.0	99.97±0.0	99.97±0.0
	7	98.17±0.1	97.91±0.1	97.32±0.7	99.83±0.0	99.77±0.0	99.56±0.1	99.96±0.0	99.96±0.0	99.96±0.0

**Table 4:** OAs, Kappas, and AAs using the proposed GhoMR-Net and other state-of-the-art methods on 10% and 20% training samples.

Training	Methods	IP			UP			SA		
		OA	Kappa	AA	OA	Kappa	AA	OA	Kappa	AA
10%	SVM	81.67±0.6	78.76±0.8	79.84±3.4	90.58±0.5	87.21±0.7	92.99±0.4	94.46±0.1	93.13±0.3	93.01±0.6
	2D-CNN	80.27±1.2	78.26±2.1	68.32±4.1	96.63±0.2	95.53±1.0	94.84±1.4	96.34±0.3	95.93±0.9	94.36±0.5
	3D-CNN	82.62±0.1	79.25±0.3	76.51±0.1	96.34±0.2	94.90±1.2	97.03±0.6	85.00±0.1	83.20±0.7	89.63±0.2
	M3D-CNN	81.39±2.6	81.20±2.0	75.22±0.7	95.95±0.6	93.40±0.4	97.52±1.0	94.20±0.8	93.61±0.3	96.66±0.5
	Two-CNN	96.71±0.1	96.10±0.1	96.16±0.1	97.71±0.1	97.62±0.1	97.45±0.2	97.12±0.3	96.98±0.2	97.00±0.2
	SENet (GMP)	97.48±0.3	97.84±0.2	97.91±0.3	97.56±0.5	97.41±0.4	97.47±0.4	98.88±0.1	98.93±0.2	99.01±0.1
	SENet (GAP)	97.62±0.3	97.91±0.2	97.88±0.3	97.53±0.6	97.48±0.5	97.52±0.5	99.11±0.2	98.89±0.2	99.06±0.2
	FuSENet	98.11±0.2	98.25±0.2	98.32±0.2	97.65±0.3	97.69±0.3	97.68±0.4	99.23±0.1	98.97±0.2	99.16±0.1
	SSRN	98.45±0.2	98.23±0.3	86.19±1.3	99.62±0.0	99.50±0.0	99.49±0.0	99.64±0.0	99.60±0.0	99.76±0.0
	HybridSN	98.39±0.4	98.16±0.5	98.01±0.5	99.72±0.1	99.64±0.2	99.20±0.2	99.98±0.0	99.98±0.0	99.98±0.0
	**GhoMR-Net**	98.64±0.2	98.45±0.3	98.00±0.8	99.75±0.0	99.67±0.0	99.33±0.1	99.98±0.0	99.98±0.0	99.98±0.0
20%	SVM	86.24±0.4	84.27±0.5	83.15±1.1	95.20±0.1	93.63±0.2	93.60±0.1	94.15±0.1	93.48±0.1	97.23±0.1
	2D-CNN	86.90±1.3	85.01±1.6	82.70±1.0	96.02±0.4	96.04±0.3	95.10±0.1	96.15±0.6	95.71±0.7	98.27±0.2
	3D-CNN	89.23±0.2	87.70±0.3	87.87±0.1	97.30±0.3	96.22±0.1	97.02±0.1	94.54±0.5	93.81±0.3	96.79±0.6
	M3D-CNN	93.67±0.1	92.70±0.3	93.60±0.6	97.41±0.2	96.05±0.6	98.22±0.1	94.92±0.3	94.40±0.1	97.28±0.2
	Two-CNN	98.73±0.2	98.71±0.2	98.73±0.2	98.72±0.3	98.40±0.2	98.45±0.2	98.13±0.4	98.01±0.2	98.10±0.2
	SENet (GMP)	98.53±0.6	98.27±0.8	97.91±1.5	99.05±0.2	98.81±0.2	98.86±0.2	99.07±0.3	99.19±0.2	99.13±0.2
	SENet (GAP)	98.76±0.5	98.43±0.7	98.20±1.0	99.36±0.1	99.20±0.1	99.30±0.1	99.50±0.1	99.55±0.1	99.40±0.1
	FuSENet	99.01±0.1	98.60±0.1	98.64±0.1	99.42±0.2	99.21±0.3	99.33±0.2	99.68±0.2	99.74±0.1	99.69±0.1
	SSRN	99.23±0.1	99.12±0.1	92.52±0.1	99.77±0.1	99.69±0.2	99.71±0.1	99.88±0.0	99.87±0.0	99.84±0.0
	HybridSN	99.47±0.1	99.40±0.1	99.38±0.1	99.86±0.1	99.82±0.0	99.71±0.1	100±0.0	100±0.0	100±0.0
	**GhoMR-Net**	99.54±0.0	99.47±0.0	99.30±0.2	99.90±0.0	99.86±0.0	99.82±0.0	99.99±0.0	99.99±0.0	99.99±0.0

**Table 5:** OAs, Kappas and AAs with lesser training samples (in %) and smaller spatial size of input data on IP, UP and SA datasets respectively.

Training Samples	Spatial Size	IP			UP			SA		
		OA	Kappa	AA	OA	Kappa	AA	OA	Kappa	AA
5%	13 × 13	95.42±0.9	94.77±1.0	84.68±5.1	99.58±0.1	99.44±0.1	99.18±0.1	99.77±0.1	99.74±0.1	99.81±0.1
	11 × 11	94.23±0.1	93.42±0.1	84.72±2.1	99.61±0.0	99.49±0.1	99.28±0.1	99.62±0.1	99.58±0.1	99.73±0.0
3%	13 × 13	89.48±1.7	87.96±2.0	73.48±2.4	99.34±0.1	99.13±0.1	98.76±0.2	99.85±0.0	99.83±0.0	99.85±0.1
	11 × 11	87.95±1.2	86.23±1.4	72.75±3.6	99.41±0.1	99.22±0.1	99.00±0.1	99.57±0.2	99.52±0.2	99.71±0.1

## References

[1] Park B., Lu R. (2015). Hyperspectral Imaging Technology in Food and Agriculture.

[2] Goodenough D.G., Chen H., Gordon P., Niemann K.O., Quinn G. Forest applications with hyperspectral imaging. Proceedings of the IEEE International Geoscience and Remote Sensing Symposium.

[3] Tusa E., Laybros A., Monnet J.M., Dalla Mura M., Barré J.B., Vincent G., Dalponte M., Feret J.B., Chanussot J. (2020). Fusion of hyperspectral imaging and LiDAR for forest monitoring. Data Handling in Science and Technology.

[4] Liang H. (2012). Advances in multispectral and hyperspectral imaging for archaeology and art conservation. Appl. Phys. A.

[5] Calin M.A., Parasca S.V., Savastru D., Manea D. (2014). Hyperspectral imaging in the medical field: Present and future. Appl. Spectrosc. Rev..

[6] Huang H., Liu L., Ngadi M.O. (2014). Recent developments in hyperspectral imaging for assessment of food quality and safety. Sensors.

[7] Ardouin J.P., Lévesque J., Rea T.A. A demonstration of hyperspectral image exploitation for military applications. Proceedings of the 10th International Conference on Information Fusion.

[8] Edelman G., Gaston E., Van Leeuwen T., Cullen P., Aalders M. (2012). Hyperspectral imaging for non-contact analysis of forensic traces. Forensic Sci. Int..

[9] Villa A., Benediktsson J.A., Chanussot J., Jutten C. (2011). Hyperspectral image classification with independent component discriminant analysis. IEEE Trans. Geosci. Remote Sens..

[10] Licciardi G., Marpu P.R., Chanussot J., Benediktsson J.A. (2011). Linear versus nonlinear PCA for the classification of hyperspectral data based on the extended morphological profiles. IEEE Geosci. Remote Sens. Lett..

[11] Bandos T.V., Bruzzone L., Camps-Valls G. (2009). Classification of hyperspectral images with regularized linear discriminant analysis. IEEE Trans. Geosci. Remote Sens..

[12] Hong D., Yokoya N., Chanussot J., Xu J., Zhu X.X. (2020). Joint and Progressive Subspace Analysis (JPSA) with Spatial-Spectral Manifold Alignment for Semi-Supervised Hyperspectral Dimensionality Reduction. arXiv.

[13] Liu H., Xia K., Li T., Ma J., Owoola E. (2020). Dimensionality Reduction of Hyperspectral Images Based on Improved Spatial–Spectral Weight Manifold Embedding. Sensors.

[14] Hong D., Yokoya N., Chanussot J., Xu J., Zhu X.X. (2019). Learning to propagate labels on graphs: An iterative multitask regression framework for semi-supervised hyperspectral dimensionality reduction. ISPRS J. Photogramm. Remote Sens..

[15] Wang Q., Li Q., Li X. (2020). A Fast Neighborhood Grouping Method for Hyperspectral Band Selection. IEEE Trans. Geosci. Remote Sens..

[16] Lorenzo P.R., Tulczyjew L., Marcinkiewicz M., Nalepa J. (2020). Hyperspectral band selection using attention-based convolutional neural networks. IEEE Access.

[17] Sun W., Peng J., Yang G., Du Q. (2020). Fast and latent low-rank subspace clustering for hyperspectral band selection. IEEE Trans. Geosci. Remote Sens..

[18] Han Z., Hong D., Gao L., Zhang B., Chanussot J. (2020). Deep Half-Siamese Networks for Hyperspectral Unmixing. IEEE Geosci. Remote Sens. Lett..

[19] Hong D., Yokoya N., Chanussot J., Zhu X.X. (2018). An augmented linear mixing model to address spectral variability for hyperspectral unmixing. IEEE Trans. Image Process..

[20] Khajehrayeni F., Ghassemian H. (2020). Hyperspectral unmixing using deep convolutional autoencoders in a supervised scenario. IEEE J. Sel. Top. Appl. Earth Obs. Remote Sens..

[21] Li W., Chen C., Su H., Du Q. (2015). Local binary patterns and extreme learning machine for hyperspectral imagery classification. IEEE Trans. Geosci. Remote Sens..

[22] Kang X., Li C., Li S., Lin H. (2017). Classification of hyperspectral images by Gabor filtering based deep network. IEEE J. Sel. Top. Appl. Earth Obs. Remote Sens..

[23] Fang L., He N., Li S., Plaza A.J., Plaza J. (2018). A new spatial–spectral feature extraction method for hyperspectral images using local covariance matrix representation. IEEE Trans. Geosci. Remote Sens..

[24] Melgani F., Bruzzone L. (2004). Classification of hyperspectral remote sensing images with support vector machines. IEEE Trans. Geosci. Remote Sens..

[25] Benediktsson J.A., Palmason J.A., Sveinsson J.R. (2005). Classification of hyperspectral data from urban areas based on extended morphological profiles. IEEE Trans. Geosci. Remote Sens..

[26] Camps-Valls G., Gomez-Chova L., Muñoz-Marí J., Vila-Francés J., Calpe-Maravilla J. (2006). Composite kernels for hyperspectral image classification. IEEE Geosci. Remote Sens. Lett..

[27] Li J., Marpu P.R., Plaza A., Bioucas-Dias J.M., Benediktsson J.A. (2013). Generalized composite kernel framework for hyperspectral image classification. IEEE Trans. Geosci. Remote Sens..

[28] Tang Y.Y., Lu Y., Yuan H. (2014). Hyperspectral image classification based on three-dimensional scattering wavelet transform. IEEE Trans. Geosci. Remote Sens..

[29] Jia S., Shen L., Li Q. (2014). Gabor feature-based collaborative representation for hyperspectral imagery classification. IEEE Trans. Geosci. Remote Sens..

[30] Chen Y., Nasrabadi N.M., Tran T.D. (2011). Hyperspectral image classification using dictionary-based sparse representation. IEEE Trans. Geosci. Remote Sens..

[31] Fang L., Li S., Kang X., Benediktsson J.A. (2014). Spectral–spatial hyperspectral image classification via multiscale adaptive sparse representation. IEEE Trans. Geosci. Remote Sens..

[32] Fang L., Wang C., Li S., Benediktsson J.A. (2017). Hyperspectral image classification via multiple-feature-based adaptive sparse representation. IEEE Trans. Instrum. Meas..

[33] Rasti B., Hong D., Hang R., Ghamisi P., Kang X., Chanussot J., Benediktsson J.A. (2020). Feature extraction for hyperspectral imagery: The evolution from shallow to deep. arXiv.

[34] Krizhevsky A., Sutskever I., Hinton G.E. ImageNet Classification with Deep Convolutional Neural Networks. Proceedings of the 25th International Conference on Neural Information Processing Systems.

[35] Deng J., Dong W., Socher R., Li L.J., Li K., Fei-Fei L. Imagenet: A large-scale hierarchical image database. Proceedings of the IEEE Conference on Computer Vision and Pattern Recognition.

[36] Simonyan K., Zisserman A. (2014). Very deep convolutional networks for large-scale image recognition. arXiv.

[37] Szegedy C., Liu W., Jia Y., Sermanet P., Reed S., Anguelov D., Erhan D., Vanhoucke V., Rabinovich A. Going deeper with convolutions. Proceedings of the IEEE Conference on Computer Vision and Pattern Recognition.

[38] He K., Zhang X., Ren S., Sun J. Deep residual learning for image recognition. Proceedings of the IEEE Conference on Computer Vision and Pattern Recognition.

[39] Huang G., Liu Z., Van Der Maaten L., Weinberger K.Q. Densely connected convolutional networks. Proceedings of the IEEE Conference on Computer Vision and Pattern Recognition.

[40] Hu J., Shen L., Sun G. Squeeze-and-excitation networks. Proceedings of the IEEE Conference on Computer Vision and Pattern Recognition.

[41] Girshick R., Donahue J., Darrell T., Malik J. Rich feature hierarchies for accurate object detection and semantic segmentation. Proceedings of the IEEE Conference on Computer Vision and Pattern Recognition.

[42] Girshick R. Fast R-CNN. Proceedings of the IEEE International Conference on Computer Vision.

[43] Ren S., He K., Girshick R., Sun J. (2016). Faster R-CNN: Towards real-time object detection with region proposal networks. IEEE Trans. Pattern Anal. Mach. Intell..

[44] Redmon J., Divvala S., Girshick R., Farhadi A. You only look once: Unified, real-time object detection. Proceedings of the IEEE Conference on Computer Vision and Pattern Recognition.

[45] Liu W., Anguelov D., Erhan D., Szegedy C., Reed S., Fu C.Y., Berg A.C. (2016). Ssd: Single shot multibox detector. European Conference on Computer Vision.

[46] He K., Gkioxari G., Dollár P., Girshick R. Mask R-CNN. Proceedings of the IEEE International Conference on Computer Vision.

[47] Badrinarayanan V., Kendall A., Cipolla R. (2017). Segnet: A deep convolutional encoder-decoder architecture for image segmentation. IEEE Trans. Pattern Anal. Mach. Intell..

[48] Long J., Shelhamer E., Darrell T. Fully convolutional networks for semantic segmentation. Proceedings of the IEEE Conference on Computer Vision and Pattern Recognition.

[49] Ronneberger O., Fischer P., Brox T. U-net: Convolutional networks for biomedical image segmentation. Proceedings of the International Conference on Medical Image Computing and Computer-Assisted Intervention.

[50] Basha S.S., Ghosh S., Babu K.K., Dubey S.R., Pulabaigari V., Mukherjee S. Rccnet: An efficient convolutional neural network for histological routine colon cancer nuclei classification. Proceedings of the 15th International Conference on Control, Automation, Robotics and Vision.

[51] Makantasis K., Karantzalos K., Doulamis A., Doulamis N. Deep supervised learning for hyperspectral data classification through convolutional neural networks. Proceedings of the IEEE International Geoscience and Remote Sensing Symposium.

[52] Hamida A.B., Benoit A., Lambert P., Amar C.B. (2018). 3-D deep learning approach for remote sensing image classification. IEEE Trans. Geosci. Remote Sens..

[53] Zhu J., Fang L., Ghamisi P. (2018). Deformable convolutional neural networks for hyperspectral image classification. IEEE Geosci. Remote Sens. Lett..

[54] Hao S., Wang W., Ye Y., Li E., Bruzzone L. (2018). A deep network architecture for super-resolution-aided hyperspectral image classification with classwise loss. IEEE Trans. Geosci. Remote Sens..

[55] Yang J., Zhao Y.Q., Chan J.C.W. (2017). Learning and transferring deep joint spectral–spatial features for hyperspectral classification. IEEE Trans. Geosci. Remote Sens..

[56] He M., Li B., Chen H. Multi-scale 3D deep convolutional neural network for hyperspectral image classification. Proceedings of the IEEE International Conference on Image Processing.

[57] Zhang H., Li Y., Jiang Y., Wang P., Shen Q., Shen C. (2019). Hyperspectral classification based on lightweight 3-D-CNN with transfer learning. IEEE Trans. Geosci. Remote Sens..

[58] Zhong Z., Li J., Luo Z., Chapman M. (2017). Spectral–spatial residual network for hyperspectral image classification: A 3-D deep learning framework. IEEE Trans. Geosci. Remote Sens..

[59] Roy S.K., Krishna G., Dubey S.R., Chaudhuri B.B. (2019). HybridSN: Exploring 3-D–2-D CNN feature hierarchy for hyperspectral image classification. IEEE Geosci. Remote Sens. Lett..

[60] Kang X., Zhuo B., Duan P. (2018). Dual-path network-based hyperspectral image classification. IEEE Geosci. Remote Sens. Lett..

[61] Yu Y., Gong Z., Wang C., Zhong P. (2017). An unsupervised convolutional feature fusion network for deep representation of remote sensing images. IEEE Geosci. Remote Sens. Lett..

[62] Song W., Li S., Fang L., Lu T. (2018). Hyperspectral image classification with deep feature fusion network. IEEE Trans. Geosci. Remote Sens..

[63] Roy S.K., Dubey S.R., Chatterjee S., Chaudhuri B.B. (2020). FuSENet: Fused squeeze-and-excitation network for spectral-spatial hyperspectral image classification. IET Image Process..

[64] Gao S., Cheng M.M., Zhao K., Zhang X.Y., Yang M.H., Torr P.H. (2019). Res2net: A new multi-scale backbone architecture. IEEE Trans. Pattern Anal. Mach. Intell..

[65] Han K., Wang Y., Tian Q., Guo J., Xu C., Xu C. GhostNet: More features from cheap operations. Proceedings of the IEEE Conference on Computer Vision and Pattern Recognition.

[66] Wei B., Shen X., Yuan Y. (2020). Remote Sensing Scene Classification Based on Improved GhostNet. Journal of Physics: Conference Series.

[67] Green R.O., Eastwood M.L., Sarture C.M., Chrien T.G., Aronsson M., Chippendale B.J., Faust J.A., Pavri B.E., Chovit C.J., Solis M. (1998). Imaging spectroscopy and the airborne visible/infrared imaging spectrometer (AVIRIS). Remote Sens. Environ..

[68] Ioffe S., Szegedy C. Batch Normalization: Accelerating Deep Network Training by Reducing Internal Covariate Shift. Proceedings of the Machine Learning Research.

[69] Lin M., Chen Q., Yan S. (2013). Network in network. arXiv.

[70] Kingma D.P., Ba J. (2014). Adam: A method for stochastic optimization. arXiv.

[71] Maaten L.v.d., Hinton G. (2008). Visualizing data using t-SNE. J. Mach. Learn. Res..

